# Obligate faunivorous megatheropod size class patterns across the Jurassic-Cretaceous Periods

**DOI:** 10.7717/peerj.21007

**Published:** 2026-04-09

**Authors:** Colin Boisvert, Jack Perkins, Cassius Morrison, Samuel J. L. Gascoigne, Thomas R. Holtz, Brian Curtice

**Affiliations:** 1 Oklahoma State University Center for Health Sciences, Tulsa, OK, United States; 2 School of Science & Technology, University of New England, Armidale, New South Wales, Australia; 3 Department of Earth Sciences, University College London, University of London, London, United Kingdom; 4 Fossil Reptiles, Amphibians and Birds Section, Natural History Museum, London, United Kingdom; 5 School of Biological Sciences, University of Aberdeen, Aberdeen, United Kingdom; 6 Department of Biology, University of Oxford, Oxford, United Kingdom; 7 Department of Geology, University of Maryland, College Park, MD, United States; 8 Department of Paleobiology, National Museum of Natural History, Washington DC, United States; 9 Arizona Museum of Natural History, Mesa, Arizona, United States

**Keywords:** Allosauroidea, Megalosauroidea, Tyrannosauroidea, Megaraptora, Theropod evolution, Theropod ecology, Ceratosauria, Median size class

## Abstract

Allosauroidea, Ceratosauria, Megalosauroidea, Megaraptora, and Tyrannosauroidea are five clades containing obligate faunivorous megatheropods. These clades included apex predators from the Early Jurassic until the end of the Cretaceous Period. Studying the timeline of median size class change for ceratosaurians, tyrannosauroids, and megaraptorans compared to the extinction of the incumbent apex predator clades, allosauroids and megalosauroids, is important regarding megatheropod guild structure. This study used the median size classes exhibited by these clades throughout the Jurassic and Cretaceous Periods, along with the relationship of the median number of missing size classes under different apex predatory regimes. We calculated size class medians for each clade during six discrete time bins. Statistical tests on the median size class data were run to identify potential significant differences and test if increases in median size class occurred after the Cretaceous Thermal Maximum (KTM). Statistical tests were run on the number of missing size classes for each type of apex predator regime to determine if previously proposed hypotheses may explain potential differences. Statistical differences were found between four pairs of clades and their respective median size classes. Median size class increased after the Cretaceous Thermal Maximum for tyrannosauroids and potentially megaraptorans, but increased before the Cretaceous Thermal Maximum for ceratosaurians and did not change afterwards. The median number of missing size classes was found to be higher in the abelisauroid, abelisauroid/megaraptoran, and tyrannosauroid ecosystems compared to allosauroid/megalosauroid ecosystems. The median number of missing size classes between allosauroid/megalosauroid environments and tyrannosauroid-dominated environments was found to be significantly different, with a higher median number of missing size classes in tyrannosauroid-dominated environments. The analysis provides support for hypotheses, such as intraclade niche partitioning or niche shifting, to explain the differences in the median number of missing size classes between abelisauroid, abelisauroid/megaraptoran, and tyrannosauroid ecosystems and allosauroid/megalosauroid ecosystems. This study implies a complex history regarding the timing of the increase in the median size class for clades that survived the Cretaceous Thermal Maximum, which requires further study.

## Introduction

The clades Allosauroidea, Ceratosauria, Megalosauroidea, Megaraptora, and Tyrannosauroidea each contain species that are considered obligate faunivorous megatheropods (theropods over 1,000 kg in mass) (Schroeder, Lyons & Smith, 2021). Various combinations of species from these five clades co-occurred at different temporal and spatial points throughout the Mesozoic (Schroeder, Lyons & Smith, 2021; Holtz, 2021).

Allosauroids and megalosauroids were the dominant apex predators in the majority of the globe from the Middle Jurassic to Early Cretaceous, until their extinction during the early Late Cretaceous (Schroeder, Lyons & Smith, 2021; Holtz, 2021). Subsequently, tyrannosaurids became the dominant predators in the majority of Laurasian ecosystems in the Late Cretaceous (Holtz, 2021). In Gondwana, following the extinction of allosauroids and megalosauroids, abelisaurids and megaraptorans became the apex predators (Lamanna et al., 2020; Holtz, 2021; Lamanna et al., 2022). Europe was inundated by a shallow sea throughout most of the Mesozoic (Csiki-Sava et al., 2015) and its geoposition produced an ecotone (*i.e*., a zone in which two distinct biological communities intermix) especially in the Late Cretaceous (Wallace, 1860; Clements, 1904; Smith, 1974; Canudo et al., 2009; Osi, Butler & Weishampel, 2010; Prieto-Marquez, 2010; Ezcurra & Agnolín, 2012; Csiki-Sava et al., 2015; Vila, Sellés & Brusatte, 2016; Sallam et al., 2018; Delcourt, 2018; Krause et al., 2019; Longrich et al., 2021; Santos-Cubedo et al., 2023; Czepiński & Madzia, 2024; Mocho et al., 2024).

Late Cretaceous Europe was similar to Gondwana, where abelisaurid ceratosaurs were the dominant predators and the only currently known clade of megatheropods (Delcourt & Iori, 2020). The extinction of Allosauroidea and Megalosauroidea during the Cenomanian–Turonian was a key transitional period for dinosaur faunas globally (Zanno et al., 2019; Nesbitt et al., 2019; Holtz, 2021; Noto et al., 2022) and may represent a series of staggered extinctions (Mannion, 2024) rather than a single cataclysmic event.

Body mass is an important metric for studying organisms (Campione & Evans, 2020; Blanco et al., 2021, 2025). However, body mass is variable for animals, so size classes help account for variable taxon masses, mass estimations, and other factors (Campione & Evans, 2020; Holtz, 2021). When discussing these five clades containing obligate faunivorous megatheropods (Ceratosauria, Megalosauroidea, Allosauroidea, Megaraptora, and Tyrannosauroidea), analyzing size class trends through time is important as one aspect of their evolutionary trajectories. As taxa from these clades coexisted with each other, understanding trends regarding size class may help with studying niche partitioning between them (Nesbitt et al., 2019; Schroeder, Lyons & Smith, 2021; Holtz, 2021; Cau, 2024; Kotevski et al., 2025). Studying size class trends can be utilized to analyze if clades such as tyrannosauroids, abelisauroids, and megaraptorans began increasing their median clade size class before the extinction of allosauroids and megalosauroids or in response to this extinction (Nesbitt et al., 2019; Lamanna et al., 2020; Holtz, 2021; Aranciaga Rolando et al., 2022; Kotevski et al., 2025; Morrison et al., 2025).

We examined median size classes for theropod species in these five clades across six time bins (from the Early Jurassic to the Late Cretaceous). Time bins used for this study relate to period boundaries or important climatic events. We also reanalyzed size class data from Holtz (2021) to discern possible statistical differences in the median number of missing size classes between environments with different apex predators present. As these five clades interacted in different ways throughout the Mesozoic, studying the median size class allows us to explore patterns related to niche partitioning and replacement. Our first hypothesis (H1) is that median size class will increase across the Cretaceous Thermal Maximum for the surviving clades due to the extinction of allosauroids and megalosauroids. Our second hypothesis (H2) is the median number of missing size classes will increase in the Coniacian-Maastrichtian due to the prevalence of niche shifting and/or dietary shifts in tyrannosaurs, intraclade niche partitioning in Abelisauridae, and potential habitat partitioning between megaraptorans and abelisaurids in latest Cretaceous South America.

### Background and assumptions

Some background information and certain assumptions were utilized for this study. The Pliensbachian-Toarcian Crisis and the Cretaceous Thermal Maximum are two important climate events due to the dinosaur faunal turnover documented (Zanno et al., 2019; Nesbitt et al., 2019; Pol et al., 2020; Burgener et al., 2023; Filippi et al., 2023; Dunhill et al., 2024; Upchurch & Chiarenza, 2024; Chiarenza, 2024; Mannion, 2024). For the late Early Jurassic, current analysis points to a faunal turnover on land and in the ocean potentially due to volcanic activity and subsequent environmental changes that led to the extinction of basal sauropodomorphs and the rise of sauropods including potentially neosauropods (Xu et al., 2018; Dunhill et al., 2018; Pol et al., 2020; Dunhill et al., 2024; Upchurch & Chiarenza, 2024).

Furthermore, while the first ceratosaurians evolved and diversified in the Early Jurassic, several other clades, including early megalosaurs, abelisaurs, and allosauroids, are currently known to have first appeared after the Pliensbachian-Toarcian Crisis (Pol & Rauhut, 2012; Rauhut, Hübner & Lanser, 2016; Rauhut & Pol, 2019; Pradelli, Pol & Ezcurra, 2024; Upchurch & Chiarenza, 2024). This combination of newly evolved predators, illustrated in the dataset as only ceratosaurs are currently reported in the Hettangian-Pliensbachian time bin and eusauropod-dominated faunas, indicates an important transition in dinosaur fauna in the Early Jurassic. As this crisis led to a change in fauna, time bins for this study should be treated as distinct. Similarly, in Europe between the Middle Jurassic and Late Jurassic, there is evidence for a theropod faunal turnover supporting constraining time bins using the period boundaries between the Middle and Late Jurassic (Rauhut, Hübner & Lanser, 2016).

Before the Cenomanian-Turonian extinctions, we see members of all five clades living in different environments, with members of megaraptorans, ceratosaurians, and tyrannosauroids generally as small to medium-sized predators and allosauroids and megalosauroids as apex predators (Zanno & Makovicky, 2013; Zanno et al., 2019; Nesbitt et al., 2019; Schroeder, Lyons & Smith, 2021; Holtz, 2021; Noto et al., 2022; Aranciaga Rolando et al., 2022; Pereyra et al., 2025a). The Cretaceous Thermal Maximum during the early Late Cretaceous (Cenomanian-Turonian) displays an important faunal turnover, including the extinction of clades such as allosauroids, megalosauroids, and diplodocoids that have been associated with climatic changes (Zanno et al., 2019; Nesbitt et al., 2019; Noto et al., 2022; Aranciaga Rolando et al., 2022; Burgener et al., 2023; Filippi et al., 2023; Pereyra et al., 2025a). This turnover saw the rise of dinosaurian clades that would dominate the remainder of the Cretaceous until the Cretaceous-Paleogene (K-Pg) Extinction, such as tyrannosaurids, neoceratopsians, hadrosaurids, and abelisaurids, and with the loss of rebbachisaurids, left titanosaurs as the sole surviving sauropod clade (Zanno et al., 2019; Nesbitt et al., 2019; Longrich et al., 2021; Noto et al., 2022; Aranciaga Rolando et al., 2022; Filippi et al., 2023). The faunas before and after the Cretaceous Thermal Maximum (Cenomanian-Turonian Stages) are distinct.

Tyrannosauroids, megaraptorans, and abelisaurs expanded into the vacated niche space after the Cenomanian-Turonian, and generally increased their size class across the clades with the alteration in theropod guild structure (Zanno et al., 2019; Nesbitt et al., 2019; Schroeder, Lyons & Smith, 2021; Holtz, 2021; Noto et al., 2022; Aranciaga Rolando et al., 2022; Mannion, 2024; Pereyra et al., 2025a; Morrison et al., 2025). The stated factors for (H2) are based on several hypotheses previously proposed or observed (Bandyopadhyay et al., 2010; Delcourt, 2018; Porfiri et al., 2018; Nesbitt et al., 2019; Novas et al., 2019; Lamanna et al., 2020; Therrien et al., 2021; Schroeder, Lyons & Smith, 2021; Longrich et al., 2021; Holtz, 2021; Lamanna et al., 2022; Davis et al., 2023; Longrich et al., 2023; Therrien et al., 2023; Delcourt et al., 2024; Ibiricu et al., 2025). Regarding potential ontogenetic niche shifting in tyrannosaurs, this idea requires further study and may be partially altered for *Tyrannosaurus rex* in the Hell Creek Formation with the resurrection of *Nanotyrannus lancensis* and the new species *Nanotyrannus lethaeus* (Longrich & Saitta, 2024; Zanno & Napoli, 2025; Griffin et al., 2025).

We made several *a priori* assumptions for this study. We assumed there was no bias in what fossils were collected from localities with regard to taxa of different body sizes or for different clades. We assumed taxa from all clades were preserved at comparable rates, with no radical anatomical differences between clades that would impact deposition. While certain formations have been known to illustrate specific preservational biases, such as the Dinosaur Park Formation or Hell Creek Formation (size-biased preservation) (Leach, Hoffman & Dodson, 2021; Brown et al., 2022), others, such as the Morrison Formation, do not show any biases (Leach, Hoffman & Dodson, 2021). With this in mind, we assumed formation preservational biases were minimal. This study focused on elucidating when the median size class changed for the tyrannosauroids, megaraptorans, and abelisauroids, as this relates to broader themes of opportunistic niche takeover *vs* evolutionary outcompetition. Further study of ecosystem-specific differences in predatory size class distribution may reveal new study questions to understand broader theropod guild structure surrounding these clades.

## Methods

A partially updated copy of an occurrence matrix designed by Krzysztof Stuchlik was provided by BC for this study. For the original matrix, we selected valid species from five clades: Allosauroidea, Ceratosauria, Megalosauroidea, Megaraptora, and Tyrannosauroidea (File S1). New species from these clades were added to the matrix as they were described. Some taxa were added or removed from our updated matrix as new information became available regarding their validity, such as *Timimus hermani* and *Bagaraatan ostromi* (Delcourt & Grillo, 2018; Słowiak, Brusatte & Szczygielski, 2024). While recent claims questioned the taxonomic status of *Ulughbegsaurus uzbekistanensis* (Sues, Averianov & Britt, 2023) as a valid carcharodontosaurian, new evidence indicates it does represent a member of this clade (Tanaka et al., 2021; Averianov & Sues, 2024; Averianov et al., 2025). However, we did not add *Ulughbegsaurus uzbekistanensis*, *Ulughbegsaurus sp*., *Yuanmouraptor jinshajiangensis*, *Khankhuuluu mongoliensis*, *Joaquinraptor casali*, *Vitosaura colozacani*, *Nanotyrannus lancensis*, and *Nanotyrannus lethaeus* due to the advanced stage of the manuscript when these taxa were described or discussed (Longrich & Saitta, 2024; Zou et al., 2025; Averianov et al., 2025; Voris et al., 2025; Ibiricu et al., 2025; Jiménez Velandia et al., 2025; Zanno & Napoli, 2025; Griffin et al., 2025). We also did not address problematic species discussed in Cau & Paterna (2025), nor removed the potentially chimeric *Protathlitis cinctorrensis* (Rauhut, Canudo & Castanera, 2025) due to the advanced stage of the manuscript. Species were assigned a single temporal value and a single continent-level landmass (File S2). Species were assigned length and mass values based on estimates made in previous works (Benson et al., 2018; Campione & Evans, 2020) and comparisons to related species, as well as size classes as defined in Holtz (2021) (Table 1) (File S2). While the holotype of *Indosaurus matleyi* appears to be missing, we gave this species a conservative size of seven meters in length based on comparison with related species.

**Table 1:** Holtz (2021) size class values used for study.

Size classes (Holtz, 2021)
1: x ≤ 10 kg
2: 11–50 kg
3: 51–100 kg
4: 101–500 kg
5: 501–1,000 kg
6: 1,001–5,000 kg
7: x > 5,000 kg

Latitude/longitude coordinates and paleocoordinates were assigned to each species based on the data available on the Palaeobiology Database (PBDB). PBDB had modern coordinates for *Tarascosaurus salluvicus*, but not paleocoordinates, so GPlates was used to calculate paleocoordinates based on the time value for this taxon (Williams et al., 2012; Scotese, 2016; Müller et al., 2018). It should be noted that the coordinates provided through GPlates for *T. salluvicus* were in a marine setting, indicative of postmortem transportation for this specimen. *Labocania aguillonae*, *Riojavenatrix lacustris*, and *Tameryraptor markgrafi* had yet to be added to PBDB during earlier rounds of manuscript review, but modern coordinates and paleocoordinates taken from the entry on their respective type localities were later modified. These modifications were done by comparing the coordinates from the type localities found with the coordinates for the entries for these species that were later added to PBDB and updating them using the entries for the specific taxa from PBDB as needed (Stromer, 1931; Viera & Torres, 1995; Martinez, 2010; Viera & Torres, 2013; Isasmendi et al., 2024; Kellermann, Cuesta & Rauhut, 2025).

### Code creation

The plots were run in RStudio (File S3). The Jurassic and Cretaceous were split into six discrete time bins: Hettangian–Pliensbachian, Toarcian–Callovian, Oxfordian–Tithonian, Berriasian–Albian, Cenomanian–Turonian, and Coniacian–Maastrichtian. The boundaries between these time bins were chosen based on important climatic events associated with faunal turnover, such as how the Cretaceous Thermal Maximum was treated as a separate time bin or period boundaries to constrain the time bins (Barclay, McElwain & Sageman, 2010; Zanno et al., 2019; Nesbitt et al., 2019; Pol et al., 2020; Burgener et al., 2023; Filippi et al., 2023; Dunhill et al., 2024). Period boundaries have been used in other large temporal scale studies, such as biogeographical analyses; hence, it is consistent to use them here regarding the Middle Jurassic, Late Jurassic, Early Cretaceous, and Latest Cretaceous to separate out the fauna and look for patterns (Brocklehurst et al., 2018). From the single temporal value assigned to species, species are placed into a time bin.

We tallied the number of theropod species from each clade for each time bin and the number of species per landmass for each clade in Excel. This data was run *via* supplementary project files in RStudio (Files S4, S5) to produce temporal and spatial plots for these clades, showing their distribution across time and space (Figs. S1, S2). We further grouped the size classes of all species of each clade per the time bin they were assigned. Then, per time bin and per clade, we calculated the median size class for each clade per time bin and double-checked them against calculations made and plotted in RStudio (Table 2) (File S6). The total median for each clade was determined by taking the median from the values for the time bins for each clade, excluding values of N/A assigned for time bins with only a single taxon occurrence for that clade (Table 2). The total active median was determined by taking the median from non-zero values in the time bins for each clade and excluding values of N/A assigned for time bins with only a single taxon occurrence for that clade as well (Table 2).

**Table 2:** Clade median size class per time bin.

Time Bin	Ceratosauria	Megalosauroidea	Allosauroidea	Megaraptora	Tyrannosauroidea
Hettangian-Pliensbachian	N/A	0	0	0	0
Toarcian-Callovian	4	5	5	0	4
Oxfordian-Tithonian	5	5	6	0	3
Berriasian-Albian	4	6	6	4	4
Cenomanian-Turonian	5	N/A	6	N/A	2
Coniacian-Maastrichtian	5	0	0	5	6
Total median (All time bins excluding N/A values)	5	5	6	0*	4
Total active median (Excluding time bins with value of zero and N/A)	5	5	6	5	4

**Note:**

The clade median size class per time bin, with N/A to indicate single taxon occurrences for that clade in that time bin. The final two rows give medians, including all non N/A time bins and then just using the time bins (all non-zero and non N/A values) fossils of that clade have been found in. The asterisk in the total median for Megaraptora (*) indicates a total median of zero, which is an artifact of the large number of time bins with zero occurrences for this clade, as discussed in the text.

Plots were produced using the R package ‘tidyverse’ (Wickham et al., 2019). The R packages ‘ggforce’, ‘ggalt’, ‘rnaturalearth’, ‘rnaturalearthdata’, and ‘tidyverse’ were used to produce the modern world map (Rudis et al., 2017; South, 2017; South, Michael & Massicotte, 2017; Wickham et al., 2019; Pedersen, 2024). Paleomaps were plotted using the R packages ‘palaeoverse’, ‘clipr’, ‘rgplates’, and ‘sf’ (Müller et al., 2018; Pebesma, 2018; Lincoln, 2022; Pebesma & Bivand, 2023; Jones et al., 2023; Kocsis et al., 2024). Data from Holtz (2021) was modified, partitioning the different formations by the main apex predators (*i.e*., allosauroid/megalosauroid, abelisauroid, abelisauroid/megaraptoran, and tyrannosauroid), along with removing two specific formations (File S7). Two formations were removed (Bissekty Fm. and Yellow Cat and Poison Strip Mbrs. of Cedar Mountain Fm.) when the analysis was run due to the lack of confirmed dominant predators from one of the clades mentioned above (File S7).

### Statistical analysis

Non-parametric statistical analysis was conducted to determine if there were differences in median size classes seen in any of the clades across time. A non-parametric test was chosen as we did not assume a normality of variance between clades. We used the R package ‘FSA’ in part to help run these non-parametric statistical tests (Ogle & Ogle, 2017). A Kruskal-Wallis test was chosen due to the heterogeneous nature of the data and the comparison of five separate study groups (File S8). Then, a Pairwise Wilcoxon rank sum test with continuity correction for comparison between pairs and a Bonferroni *post hoc* test were conducted on the statistics to determine which, if any, specific relationships between clades and size class were significantly different. A parametric and non-parametric test (one-way ANOVA and Kruskal-Wallis test, respectively) were also conducted on the modified (Holtz, 2021) data (File S7). We ran these tests based on the same methodology as the aforementioned study to determine if there was a statistical difference between specific apex predator environments and the median number of missing size classes, followed by *post hoc* tests to determine any specific statistically significant pairs between the different apex predator-ruled ecosystems. The *post hoc* tests used were Tukey multiple pairwise comparisons and a pairwise T-test with BH correction for the one-way analysis of variance (ANOVA) and a Pairwise Wilcoxon rank sum test, and a Bonferroni *post hoc* test for the Kruskal-Wallis test.

## Results

Temporal diversity, defined here as the number of species across each time bin per clade, showed a steady increase in the number of species for ceratosaurs before a sudden spike in the Coniacian-Maastrichtian Ages (Fig. S1). Tyrannosauroids display a similar trend, though their species count drops in the Cenomanian-Turonian Ages (Fig. S1). Megalosauroidea shows a bimodally peaked distribution during the Toarcian–Callovian and Berriasian–Albian Ages (Fig. S1). Allosauroidea’s distribution is similar to a normal bell curve with peaks at the Oxfordian-Tithonian and Berriasian–Albian Ages (Fig. S1). Megaraptora showed a reduced spike in the number of species during the Coniacian-Maastrichtian Ages compared to Ceratosauria and Tyrannosauroidea, but they still show a peak during this time bin (Fig. S1). Spatial diversity, defined here as the number of species across the landmasses per clade, indicated that Ceratosauria had the widest distribution, while Megaraptora had the smallest distribution (Fig. S2). Allosauroidea and Megalosauroidea showed the same spatial distribution by landmass with variation in the number of species (Fig. S2). As for total clade spatial distribution split by time bin, Tyrannosauroids are the most northern clade geographically, using the modern coordinates, while Megaraptora are the most southern clade geographically, using the modern coordinates (Fig. S3).

**Figure 1:**
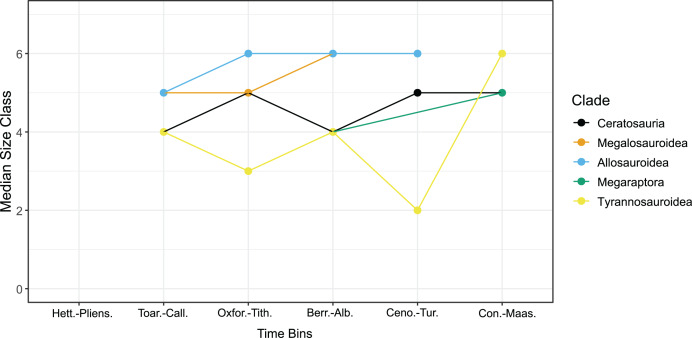
Median size class for clades per time bin. The median size class for each clade across the Jurassic and Cretaceous Periods. No valid taxa found from strata within a time bin, or that the clade was extinct during that time bin, were excluded from the plot, as the value would have been zero. Similarly, single taxon occurrences for clades within a time bin marked on the supplementary file as NA were also excluded from the plot. Abbreviations: Hett.-Pliens., Hettangian-Pliensbachian Ages; Toar.-Call., Toarcian-Callovian Ages; Oxfor.-Tith., Oxfordian-Tithonian Ages; Berr.-Alb., Berriasian-Albian Ages; Ceno.-Tur., Cenomanian-Turonian Ages; Con.-Maas., Coniacian-Maastrichtian Ages.

**Figure 2:**
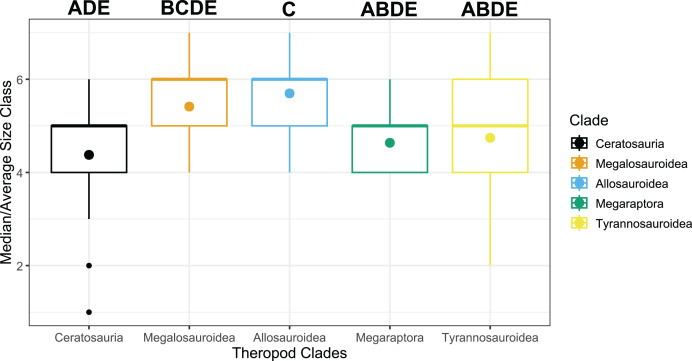
Median clade size class box plot. The median size class for each clade across time is represented by the bold line representing the median size class, a bold dot in the same clade color for the mean size class for each clade, and upper and lower quartiles. The letters above each clade relate to those pairs of clades with a significant difference between their median size classes *vs* those that were not significant. Different letters represent a significant difference in the median size classes between pairs of clades, while the same letters indicate no significant difference. For example, A in Ceratosauria and Tyrannosauroidea indicates a pair that is not significantly different, while different letters between a pair, such as A and B in Ceratosauria and Megalosauroidea, respectively, indicate a pair exhibiting a significant difference between their median size classes.

Tyrannosauroids exhibit a median size class between three to four (51–100 kg, 101–500 kg) from the Toarcian-Albian Ages (Table 2, Fig. 1). There is a decrease in tyrannosauroid median size class during the Cenomanian-Turonian to a median of size class two (11–50 kg) and then an increase to a median of size class six (1,001–5,000 kg) in the Coniacian-Maastrichtian Ages (Table 2, Fig. 1). Conversely, allosauroids start with a median size class of five (501–1,000 kg) in the Toarcian-Callovian before a consistent median size class six (1,001–5,000 kg) in the Oxfordian to the Turonian Age when they go extinct (Table 2, Fig. 1). Without the time bins, the overall median size class using all species in a clade for megalosauroids and allosauroids was six (1,001–5,000 kg), and overall median size class for tyrannosauroids, ceratosaurs, and megaraptorans was five (501–1,000 kg) (Fig. 2). This differs slightly specifically from the active median size class for megalosauroids and tyrannosauroids that uses the time bins as megalosauroids have an active median size class of five (501–1,000 kg), and tyrannosauroids have an active median size class of four (101–500 kg).

Megalosauroids display a more gradual change but similar results to allosauroids with median size class five (501–1,000 kg) in the Toarcian-Tithonian Ages in the Jurassic, before a median size class six (1,001–5,000 kg) in the Berriasian-Albian Ages (Table 2, Fig. 1). It is currently unclear what megalosauroid median size class is in the Cenomanian-Turonian Ages as this bin currently features a single taxon occurrence for this clade where only one taxon is detailed from this time bin in the data set for this clade meaning this is not a true median value (Table 2, Fig. 1). Ceratosaurians also contain a single taxon occurrence in the Hettangian–Pliensbachian Ages meaning this is not a true median, but then ceratosaurians exhibit a median size class of four (101–500 kg) from the Toarcian-Callovian Ages then an increase to a median size class of five (501–1,000 kg) in the Oxfordian-Tithonian, and repeat this pattern of median size class four and five for the Berriasian-Albian and Cenomanian-Turonian Ages respectively (Table 2, Fig. 1). Ceratosaurians then have a median size class of five (501–1,000 kg) in the Coniacian-Maastrichtian Ages (Table 2, Fig. 1). Megaraptorans show a median of size class four (101–500 kg) for the Berriasian-Albian Ages and contain a single taxon occurrence for the Cenomanian-Turonian time bin meaning this is not a true median (Table 2, Fig. 1). This is followed by an increase to a median size class of five (501–1,000 kg) in the Coniacian-Maastrichtian (Table 2, Fig. 1).

Regarding the total median for clades *vs* total active median, tyrannosauroids showed a total median and active total median of size class four (Table 2). Allosauroids displayed a total median and a total active median of size class six (Table 2). Megalosauroids and ceratosaurians showed size class five for total median and total active median size class (Table 2). Megaraptorans show a total median of size class zero, but this is not an accurate total median, but rather due to the smaller sample size of taxa for this clade, with a total active median of size class five (Table 2).

The Kruskal-Wallis test reported a significant difference among size class *vs* clades (χ^2^ = 28.651, df = 4, *p* = 9.201e−06). Furthermore, when the Pairwise Wilcoxon rank sum test was conducted, five specific pairs were found to have significant *p*-values (Table 3). These pairs were Ceratosauria-Megalosauroidea, Ceratosauria-Allosauroidea, Megalosauroidea-Megaraptora, Allosauroidea-Megaraptora, and Allosauroidea-Tyrannosauroidea. When the Bonferroni test was run, only four pairs had significant adjusted *p*-values (Table 4). These were Ceratosauria-Megalosauroidea, Ceratosauria-Allosauroidea, Allosauroidea-Megaraptora, and Allosauroidea-Tyrannosauroidea. As this test only recovered four significant pairs *vs* five, we decided to conservatively go with these four pairs and exclude Megalosauroidea-Megaraptora from further discussion. The Kruskal-Wallis test indicates there is a statistically significant difference in the median size classes between these five clades. More specifically, the *post hoc* tests indicate there are significant differences in their median size classes between these specific four pairs of clades. The results regarding allosauroids and megaraptorans and allosauroids and tyrannosaurids support (H1), as we found an increase in median size class for these clades after the extinction of allosauroids. We tentatively include megaraptorans despite the lack of a median size class in the Cenomanian-Turonian, looking at the data in the Berriasian-Albian. However, our results regarding allosauroids and ceratosaurians and megalosauroids and ceratosaurians do not support (H1) as no increase in median size class was seen after the Cretaceous Thermal Maximum, with the increase happening between the Berriasian-Albian and Cenomanian-Turonian time bins.

**Table 3:** *Post hoc* Pairwise Wilcoxon rank sum test for size class *vs* clades.

	Ceratosauria	Megalosauroidea	Allosauroidea	Megaraptora
Megalosauroidea	**0.0027**	–	–	–
Allosauroidea	**3.6e−05**	0.2850	–	–
Megaraptora	0.9632	**0.0183**	**0.0018**	–
Tyrannosauroidea	0.2924	0.0930	**0.0064**	0.5884

**Note:**

The Pairwise Wilcoxon rank sum test *post hoc* results of which pairs of clades had statistical differences between their median size classes with significant *p*-values in bold.

**Table 4:** Bonferroni *post hoc* test for size class *vs* clades.

Comparison pairs	Z value	*p*-value unadjusted	*p*-value adjusted
Allosauroidea–Ceratosauria	4.6883749	2.753832e−06	**2.753832e−05**
Allosauroidea–Megalosauroidea	1.0754156	2.821887e−01	1.000000e+00
Ceratosauria–Megalosauroidea	−3.3158995	9.134866e−04	**9.134866e−03**
Allosauroidea–Megaraptora	3.1250249	1.777900e−03	**1.777900e−02**
Ceratosauria–Megaraptora	0.1459865	8.839320e−01	1.000000e+00
Megalosauroidea–Megaraptora	2.2994960	2.147679e−02	2.147679e−01
Allosauroidea–Tyrannosauroidea	3.2201535	1.281220e−03	**1.281220e−02**
Ceratosauria–Tyrannosauroidea	−1.3176104	1.876341e−01	1.000000e+00
Megalosauroidea–Tyrannosauroidea	1.9898758	4.660462e−02	4.660462e−01
Megaraptora–Tyrannosauroidea	−0.9559247	3.391103e−01	1.000000e+00

**Note:**

The Bonferroni *post hoc* results of which pairs of clades had statistical differences between their median size classes with the significant *p*-values in bold.

**Figure 3:**
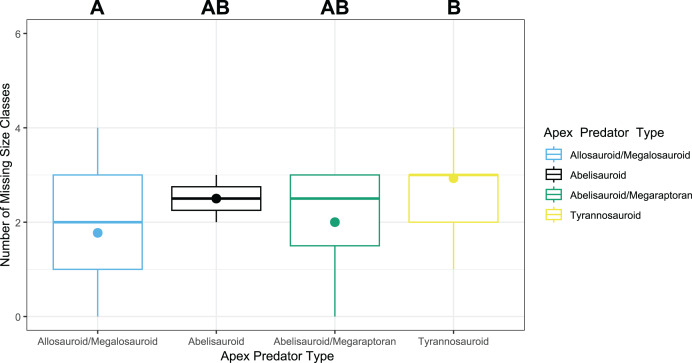
Missing size class box and whisker plot. The median missing number of size classes in different formations dominated by allosauroid/megalosauroids, abelisauroids, abelisauroid/megaraptorans, and tyrannosauroids, represented by the bold line with each clade and the mean number of missing size classes for each clade by a dot of the same clade color. The letters above each clade relate to those pairs of clades with a significant difference between their median number of missing size classes *vs* those that were not significant. Different letters represent a significant difference in the median number of missing size classes between two apex predatory environments, while the same letters indicate no significant difference. The same letters (AB in abelisauroid and abelisauroid/megaraptoran environments or A in allosauroid/megalosauroid and A in abelisauroid environments) indicates pairs that are not significantly different, while different letters between a pair (A and B in allosauroid/megalosauroid and tyrannosauroid environments, respectively) indicate a significant difference. This figure and the dataset are derived from Holtz (2021).

The median number of missing size classes per environments dominated by the four apex predatory clade groups (allosauroids/megalosauroids, abelisauroids, abelisauroid/megaraptorans, and tyrannosauroids) indicated allosauroid/megalosauroid-dominated environments exhibited the lowest median of two missing size classes. Abelisauroid and abelisauroid/megaraptoran-dominated environments exhibited a median of two and a half, and tyrannosauroids had the highest median of three missing size classes similar to results from Holtz (2021) (Fig. 3). The mean number of missing size classes for allosauroids/megalosauroids, abelisauroids, abelisauroid/megaraptorans, and tyrannosauroids environments was lower than the median for every environment except abelisauroid-dominated environments, where it was the same (Fig. 3). The one-way ANOVA test for the Holtz (2021) missing size classes *vs* predator type found in that formation data (File S7) found F = 5.497, df = 3, and *p* = 0.00231. As this F value is high, there is more variation between the sample means of the number of missing size classes of different apex predator ecosystems (ex., allosauroid/megalosauroid and tyrannosauroid ecosystems) compared to the variation between formations within the same type of ecosystem (ex., tyrannosauroid and tyrannosauroid ecosystems). The *p*-value is also below 0.05. *Post Hoc* methods using Tukey multiple pairwise comparisons and pairwise T-test with Benjamini-Hochberg adjustment method both point towards the only significant pair being tyrannosauroids-allosauroids/megalosauroids with adjusted *p*-values of 0.0011850 and 0.0013, respectively (Tables 5 and 6). However, as this data potentially contains a non-normality of variance between ecosystems, we must be cautious of these results from the parametric tests.

**Table 5:** Tukey multiple pairwise comparisons for number of missing size classes *vs* ecosystem type.

Apex predator type	Difference	95% Confidence interval lower bound	95% Confidence interval upper bound	Adjusted *p*-value
Abelisauroid-Allosauroid/Megalosauroid	0.7272727	−1.2904291	2.744975	0.7747128
Abelisauroid/Megaraptoran-Allosauroid/Megalosauroid	0.2272727	−1.2577156	1.712261	0.9771613
Tyrannosauroid-Allosauroid/Megalosauroid	1.1583072	0.3858891	1.930725	**0.0011850**
Abelisauroid/Megaraptoran-Abelisauroid	−0.5000000	−2.8659651	1.865965	0.9432459
Tyrannosauroid-Abelisauroid	0.4310345	−1.5662713	2.428340	0.9398723
Tyrannosauroid-Abelisauroid/Megaraptoran	0.9310345	−0.5261203	2.388189	0.3365821

**Note:**

The Tukey *post hoc* results of which clades showed a difference in the mean number of missing size classes in their respective apex predatory environments using data from Holtz (2021). Significant *p*-values are in bold.

**Table 6:** Pairwise T-test with BH correction for number of missing size classes *vs* ecosystem type.

	Allosauroid/Megalosauroid	Abelisauroid	Abelisauroid/Megaraptoran
Abelisauroid	0.6864	–	–
Abelisauroid/Megaraptoran	0.6864	0.6864	–
Tyrannosauroid	**0.0013**	0.6864	0.2880

**Note:**

The pairwise T-test *post hoc p*-values of which clades showed a difference in the mean number of missing size classes in their respective apex predatory environments using data from Holtz (2021). Significant *p*-values are in bold.

The Kruskal-Wallis reported a significant difference among the number of missing size classes *vs* apex predator environments (χ^2^ = 12.186, df = 3, *p* = 0.006772). Both a Pairwise Wilcoxon rank sum test and a Bonferroni test were conducted for this data, and only one pair was found with a significant *p*-value (Tables 7 and 8). This pair was the same as with the parametric data, tyrannosauroids-allosauroids/megalosauroids, with an adjusted *p*-value of 0.0043 and 0.003500551, respectively (Tables 7 and 8). As these tests were non-parametric and account for potential non-normal variation between ecosystems, the significant *p*-values do indicate there is a significant difference in the median number of missing size classes between different apex predator ruled ecosystems, and specifically a higher median number of missing size classes in tyrannosauroid compared to allosauroid/megalosauroid-ecosystems. This indicates these results support the second hypothesis (H2).

**Table 7:** *Post hoc* Pairwise Wilcoxon rank sum test for number of missing size classes *vs* ecosystem type.

	Allosauroid/Megalosauroid	Abelisauroid	Abelisauroid/Megaraptoran
Abelisauroid	0.6721	–	–
Abelisauroid/Megaraptoran	0.8222	1.0000	–
Tyrannosauroid	**0.0043**	0.6721	0.5161

**Note:**

The Pairwise Wilcoxon rank sum test *post hoc p*-values of which clades showed a difference in the median number of missing size classes in their respective apex predatory environments using data from Holtz (2021). Significant *p*-values are in bold.

**Table 8:** Bonferroni *post hoc* Test for number of missing size class *vs* ecosystem type.

Comparison pairs	Z value	*p*-value unadjusted	*p*-value adjusted
Abelisauroid–Abelisauroid/Megaraptoran	0.2737489	0.7842776194	1.000000000
Abelisauroid–Allosauroid/Megalosauroid	0.6517252	0.5145784321	1.000000000
Abelisauroid/Megaraptoran–Allosauroid/Megalosauroid	0.4493685	0.6531658664	1.000000000
Abelisauroid–Tyrannosauroid	−0.6716633	0.5017980401	1.000000000
Abelisauroid/Megaraptoran–Tyrannosauroid	−1.3651243	0.1722139915	1.000000000
Allosauroid/Megalosauroid–Tyrannosauroid	−3.4392050	0.0005834252	**0.003500551**

**Note:**

The Bonferroni *post hoc p*-values of which clades showed a difference in the median number of missing size classes in their respective apex predatory environments using data from Holtz (2021). Significant *p*-values are in bold.

**Figure 4:**
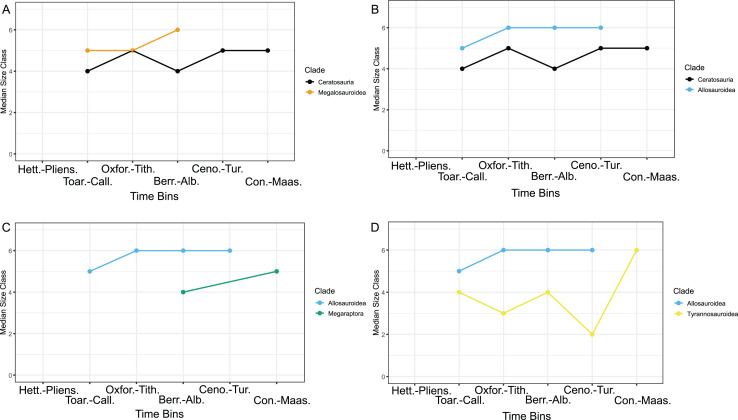
Median size class across time bins for different specific clades. Specific pairs that contained significant differences in their median size classes. (A) Ceratosaurs and megalosauroids. This plot illustrates the median size class across time bins between the two clades, Ceratosauria and Megalosauroidea, which had statistical differences in their median size classes between these clades as shown in Tables 3, 4. (B) Ceratosaurs and allosauroids. This plot illustrates the median size class across time bins between the two clades, Ceratosauria and Allosauroidea, which had statistical differences in their median size classes between these clades as shown in Tables 3, 4. (C) Allosauroids, and megaraptorans. This plot illustrates the median size class across time bins between the two clades, Allosauroidea and Megaraptora, which had statistical differences in their median size classes between these clades as shown in Tables 3, 4. (D) Allosauroids and tyrannosauroids. This plot illustrates the median size class across time bins between the two clades, Allosauroidea and Tyrannosauroidea, which had statistical differences in their median size classes between these clades as shown in Tables 3, 4. Values of zero, which represented times with no taxa or when the specific clade was extinct, or single taxon occurrences marked in the supplementary file as NA for a clade within a time bin, were excluded from the plot. Abbreviations: Hett.-Pliens., Hettangian-Pliensbachian Ages; Toar.-Call., Toarcian-Callovian Ages; Oxfor.-Tith., Oxfordian-Tithonian Ages; Berr.-Alb., Berriasian-Albian Ages; Ceno.-Tur., Cenomanian-Turonian Ages; Con.-Maas., Coniacian-Maastrichtian Ages.

The median size class for these clades per the six time bins was also plotted for comparison (Fig. 4). Ceratosaurian median size class remains at size class five once allosauroids and megalosauroids go extinct whereas the other two clades experience an increase in median size class with the more drastic increase in Tyrannosauroidea (Fig. 4). Based on the current statistical analyses, we accept aspects of (H1) as discussed above while having to reject others, and we fully accept (H2). Using the Kruskal-Wallis test and *post hoc* tests, four pairs of theropod clades that displayed statistical differences in their medial size classes were found. Two clades increased their median size class across the KTM (tyrannosauroids and potentially megaraptorans), while ceratosaurians appear to increase before the KTM and then exhibit the same median size class from the Cenomanian-Maastrichtian. Also, there was found to be an increase in the median number of missing size classes for the post-KTM apex predator ruled ecosystems compared to allosauroid/megalosauroid ecosystems. More specifically, one pair (allosauroid/megalosauroid and tyrannosauroid ecosystems) was identified by *post hoc* tests that displayed a significant difference in the median number of missing size classes. The results of the statistical tests for the Holtz (2021) data were confirmed using the R program PAST 4.15 (2023) (File S9) (Hammer, Harper & Ryan, 2001).

## Discussion

Farlow & Pianka (2002) demonstrated that diverse predatory guilds can coexist through niche partitioning, with size, morphology, and habitat use being three of the major facets of specialization, and sympatric predators often differed in at least one of these three facets. Our data indicates that differences in median size class between specific pairs require further study to determine the direction of differences. The difference between median size class and different clades is especially prevalent after the Cretaceous Thermal Maximum in the Cenomanian-Turonian (Figs. 1, 4) (Table 2). Therefore, high interclade obligate faunivorous megatheropod guild diversity may have been lessened by the extinction of allosauroids.

Body size is a major functional ecological trait heavily influencing an animal’s ecology (Blanco et al., 2021, 2025). The significant differences in median size class illustrated by our results point to potential niche partitioning between these five clades. In contemporaneous taxa, two similarly sized co-occurring species may differ in morphology (Farlow & Pianka, 2002). Our results indicate this strategy was utilized by carcharodontosaurid allosauroids and spinosaurid megalosauroids, as no significant difference in the median size class between allosauroids and megalosauroids was found (Fig. 2) (Tables 3, 4) (Beevor et al., 2021; Fabbri et al., 2022). Two contemporaneous taxa that share similar morphology may differ in size classes, such as allosauroids and some basal tyrannosauroids, allosauroids and ceratosaurians, or allosauroids and megaraptorans seen in our results that display a significant difference in median size class (Figs. 1, 2, 4B–4D) (Tables 2–4) (Farlow & Pianka, 2002; Zanno & Makovicky, 2013; Zanno et al., 2019; Foster, 2020; Holtz, 2021; Noto et al., 2022). Two contemporaneous taxa with similar morphology and comparable size may differ in preferred habitat or use of the same habitat (Farlow & Pianka, 2002). Our results illustrate that this strategy may have been utilized by allosauroids and megalosauroids or megaraptorans and ceratosaurians, which do not show significant differences in median size class between clades of either pair (Fig. 2) (Tables 3, 4) (Bakker & Bir, 2004; Rauhut, Hübner & Lanser, 2016; Beevor et al., 2021; Lamanna et al., 2022; Fabbri et al., 2022). Ceratosaurs and megalosauroids have the same median size class in the Oxfordian-Tithonian Ages (Figs. 1, 4A) (Table 2). Hypotheses regarding niche or morphological differentiation, or potential partitioned habitats, may help explain this specific trend, whereas the general results indicate differentiation in size class based on the significant difference in size class between megalosauroids and ceratosaurs (Farlow & Pianka, 2002; Bakker & Bir, 2004; Evers & Wings, 2020).

All four pairs surround one of the two incumbent apex predatory clades, mostly around Allosauroidea and one of the new clades. These pairs exhibit a shift in median size class with regard to Megaraptora and Tyrannosauroidea and a change in apex predator clade after the Cenomanian-Turonian Ages, which surround the Cretaceous Thermal Maximum (Figs. 1, 4) (Table 2) (Huber et al., 2018; Schroeder, Lyons & Smith, 2021; Holtz, 2021; Burgener et al., 2023). Tyrannosauroids decrease median size class from four down to two and then increase to six across the Berriasian-Maastrichtian Ages (Table 2). Megaraptorans increase from four to five across the same time bins; meanwhile, ceratosaurians increase from size class four in the Berriasian-Albian Ages to size class five during the Cretaceous Thermal Maximum and remain at a median size class of five in the Coniacian-Maastrichtian Ages (Table 2). This jump in median size class for two of the clades and a burst in species count may have occurred as these clades took over niche space that was once occupied by Allosauroidea and Megalosauroidea or expanded into new niche space in the case of Ceratosauria (Figs. 1, 4, S1) (Table 2) (Delcourt, 2018; Pereyra et al., 2025a, 2025b). These clades may have also experienced these bursts due to key adaptations, such as some traits exemplified in the tyrannosaur bauplan earlier in their evolution (Nesbitt et al., 2019) that allowed for incumbent replacement of the current apex predatory clades as described in Rosenzweig & McCord (1991).

Ceratosauria is a clade that occupied a diverse variety of niches and potential diets (Delcourt, 2018; de Souza et al., 2021). *Ceratosaurus* has been hypothesized to be a potential piscivore (Bakker & Bir, 2004; Delcourt, 2018). Meanwhile, other ceratosaurs have been found that were edentulous or had protruding teeth, such as *Limusaurus* and *Masiakasaurus* (Delcourt, 2018). The megalosaurid *Poekilopleuron* was discovered with fish remains in its stomach, an association also more recently reported for *Dubreuillosaurus valesdunensis* (Allain, 2005). These two discoveries suggest that piscivory was potentially plesiomorphic for Megalosauroidea, rather than an autapomorphy of Spinosauridae (Allain, 2005). Additionally, *Eustreptospondylus oxoniensis* has been suggested to be piscivorous based on skull morphology (Sadleir, Barrett & Powell, 2006). As megalosaurids have been found in a variety of habitats, it has been suggested that this clade explored different niches compared to sympatric theropods like allosauroids and ceratosaurs (Razzolini et al., 2016; Evers & Wings, 2020). This similarity in potential broad niche or habitat occupation may have led to interclade competition and explain the significant difference in median size class between ceratosaurs and megalosauroids seen in our results (Figs. 1, 4A) (Tables 2–4) (Farlow & Pianka, 2002).

Additionally, the spike in megalosauroid species compared to other clades in the Toarcian–Callovian Ages may be explained *via* sampling bias and possibly habitat preference (Rauhut, Hübner & Lanser, 2016). During most of the Mesozoic, including the Middle Jurassic, Europe was an island archipelago; this spike may be affected by a sampling bias due to both Europe having nearly 200 years of paleontological exploration in addition to megalosauroids having a potential preference of coastal habitats, while allosauroids preferred more inland habitats (Rauhut, Hübner & Lanser, 2016). A Middle Jurassic site in Portugal shows paleoethological evidence for megalosaurids crossing a tidal flat and feeding on aquatic fauna, as the unique pattern of footprints present is inconsistent with migrating or milling animals (Razzolini et al., 2016). Several other earlier branching megalosaurids have been discovered in coastal habitats, with little evidence of post-mortem transportation (Allain, 2005; Sadleir, Barrett & Powell, 2006). Coupled with evidence from stomach contents, this suggests that these taxa spent much of their time in these coastal environments. This potential habitat preference may also have helped megalosauroids avoid competition with ceratosaurs, as per Farlow & Pianka (2002).

It is currently unknown whether the extinction of megalosauroids and allosauroids occurred simultaneously across the globe or was staggered, but it is currently thought that the last refugium for these two clades was Asia (Brusatte et al., 2009, 2010; Hone, Xu & Wang, 2010; Canale et al., 2022). Megalosauroids and allosauroids seem to niche partition in many of the environments they are found in together, such as *Allosaurus* and *Torvosaurus* or *Carcharodontosaurus* and *Spinosaurus* (Bakker & Bir, 2004; Rauhut, Hübner & Lanser, 2016; Arden et al., 2019; Evers & Wings, 2020). It seems that when allosauroids and megalosauroids disappear, they are quickly replaced by other clades as apex predators (Schroeder, Lyons & Smith, 2021; Holtz, 2021; Fabbri et al., 2022; Pereyra et al., 2025b). After the turnover, intraclade niche partitioning hypothesized in tyrannosaurids and ceratosaurians is seen, and size class is not statistically different between any pairs of the surviving clades (Nesbitt et al., 2019; Therrien et al., 2021; Schroeder, Lyons & Smith, 2021; Longrich et al., 2021; Holtz, 2021). As the surviving clades did not have any statistical differences in their median size class between clades (Tables 3, 4), this suggests these clades were not influencing each other’s median size class with less interclade niche partitioning. This lack of difference in median size class between the surviving clades, their spatial distribution (Fig. S3), and current literature implies a slightly more isolated series of Late Cretaceous ecosystems (Schroeder, Lyons & Smith, 2021; Holtz, 2021; Longrich et al., 2023; Delcourt et al., 2024; Upchurch & Chiarenza, 2024).

It has been hypothesized that some theropod clades, including allosauroids, ceratosaurians, and megalosauroids, may have favored sauropods as prey items (Schroeder, Lyons & Smith, 2021; Lei et al., 2023). Climate modeling shows that sauropods may have been restricted to subtropical, lower latitude environments (Chiarenza et al., 2022; Upchurch & Chiarenza, 2024). Our distribution data indicate that Allosauroidea, Ceratosauria, and Megalosauroidea may have analogously been restricted to lower latitudes throughout the Jurassic and Cretaceous periods (Fig. S3), potentially as a function of their proposed potential preference for sauropods as prey items. However, several theropod specimens from high paleolatitude stratigraphic units in Russia and Australia have recently been referred to the ceratosaurian clade Noasauridae and the allosauroid clade Carcharodontosauria (Birch, Smith & Bell, 2020; Poropat et al., 2020; Averianov et al., 2024; Kotevski et al., 2025). Noasaurids are generally small-bodied (File S2) and occasionally herbivorous (Wang et al., 2017), while Kotevski et al. (2025) found two carcharodontosaurian tibiae from the Lower Cretaceous of Australia to represent either juvenile or subadult individuals or aberrantly small members of an otherwise macropredatory clade. As a result, the inferences that can be made on the basis of these high-latitude ceratosaurians and allosauroideans are potentially limited. Contrastingly, Tyrannosauroidea and Megaraptora do not appear to have been latitudinally restricted (Fig. S3) (Benson et al., 2012; Xu et al., 2012; Fiorillo & Tykoski, 2014; Kotevski et al., 2025).

A relationship of generally smaller tyrannosauroids and larger allosauroids is seen throughout the Late Jurassic to Early Cretaceous and into the early Late Cretaceous (Zanno & Makovicky, 2011, 2013; Nesbitt et al., 2019; Schroeder, Lyons & Smith, 2021; Holtz, 2021). In particular, it appears that top-down pressure from carcharodontosaurids may have directly impacted the ability of tyrannosauroids to expand into apex predator niches. While tyrannosauroids clearly showed a capability for achieving large size early in their evolutionary history, as evidenced by the anomalously large basal taxa *Sinotyrannus kazuoensis and Yutyrannus huali*, these taxa achieved their large size in a relatively cold and remote ecosystem apparently devoid of other megatheropod clades (Amiot et al., 2011; Xu et al., 2012). Tyrannosauroids such as *Yutyrannus* and *Sinotyrannus* may have evolved larger sizes earlier in their evolutionary history in these high-latitude localities in the absence of incumbent apex predatory theropod clades like allosauroids and megalosauroids (Xu et al., 2012). This differentiation in size class may relate to competition over niche space, as tyrannosauroids show an immediate increase in median size class and potential niche replacement after the extinction of allosauroids and in the absence of one present in the ecosystem, tyrannosauroids filled the niche as large predators (Figs. 1, 4D) (Tables 2–4) (Xu et al., 2012). Cau (2024) illustrates a marked change in community composition between tyrannosauroid and non-tyrannosauroid ecosystems in the northern hemisphere and provides some evidence for size class niche partitioning and remodeling, along with changes in theropod diversity. This implies the statistical differences between allosauroid and tyrannosauroid median size classes from this study (Tables 3, 4) may be linked to potential competition between these clades.

The incumbent presence of allosauroids may have prevented multiple clades from achieving true apex predator status. After the apparent extinction of carcharodontosaurids at the Cenomanian-Turonian boundary, while abelisaurids and megaraptorans were able to step into the void in Gondwana and western Europe, the niche of dominant predators in Asian and North American ecosystems would be filled by tyrannosauroids (Zanno et al., 2019; Nesbitt et al., 2019; Schroeder, Lyons & Smith, 2021; Longrich et al., 2021; Holtz, 2021; Aranciaga Rolando et al., 2022; Davis et al., 2023; Pereyra et al., 2025a, 2025b).

The Northern Hemisphere (Asia and North America) differed greatly in the ecological response to the Cenomanian-Turonian turnover from the Southern Hemisphere, as clades went extinct (Nesbitt et al., 2019; Brownstein, 2021; Davis et al., 2023). While some elaphrosaurines and non-furileusaurian abelisaurids suffered at least regional extinctions during the Cretaceous Thermal Maximum, Ceratosauria continued to diversify until the K-Pg, becoming apex predators (Delcourt, 2018; Holtz, 2021). Our analysis even indicates Ceratosauria as a clade increased their median size class during the Cretaceous Thermal Maximum before it stabilized, though this may be due to different factors such as niche exploration, a phylogenetic trend for decreasing size in noasaurids or morphological constraints on the abelisaur bauplan (Table 2, Figs. 4A, 4B) (Delcourt, 2018; Pereyra, Pérez & Méndez, 2025). Further new evidence from the Ibero-Armorican landmass indicates that abelisaurs may have already evolved into apex predators by the Cenomanian there (Isasmendi & Malafaia, 2025). Our results regarding the timing of the increase in ceratosaurian median size class would potentially support this hypothesis. It is unclear if and/or how much this trend seen in the Ibero-Armorican landmass pertains to the rest of the globe and requires further study.

Ceratosaurians appear to be not as restricted in size by the presence of allosauroids as tyrannosauroids, based on the relative differences in median size class (Figs. 1, 4B, 4D) (Table 2). However, ceratosaurians were potentially prevented by allosauroids and megalosauroids from reaching the top of the food web until after the Cenomanian-Turonian Faunal Turnover and may have occupied a different niche, as our results show a significant difference in median size class between allosauroids and ceratosaurians and megalosauroids and ceratosaurians (Figs. 1, 2, 4A, 4B) (Tables 2–4) (Motta et al., 2016). New research illustrates that abelisaurid size (using body length) was constrained not increasing after the extinction of carcharodontosaurids, and noasaurids exhibit a phylogenetic trend of decreasing body size (Pereyra, Pérez & Méndez, 2025). This potential stagnation of size for abelisaurids was further exhibited with a trend of low disparity in body length for Late Cretaceous abelisaurids and may be explained by their specialized feeding behavior (Pereyra, Pérez & Méndez, 2025). As for noasaurids, the trend of decreasing body size may have reduced niche overlap with other predators (Pereyra, Pérez & Méndez, 2025). While utilizing a different metric to study body size, the trend of body size stagnation is also expressed in our results with no increase in median size class for ceratosaurs between the Cenomanian-Turonian and Coniacian-Maastrichtian (Table 2) (Figs. 1, 4A, 4B). Our results may be explained in part by the stagnation of growth and decreasing body size seen in the relevant subclades of ceratosaurians (Pereyra, Pérez & Méndez, 2025), and this contrasts with megaraptorans and tyrannosauroids, who experience an increase in median size class after the extinction of allosauroids and megalosauroids (Table 2) (Figs. 1, 4A, 4B). After the extinction of allosauroids and megalosauroids, it is currently unclear if ceratosaurs fully filled this vacated niche.

There is some evidence that megaraptorans would have had different predatory habits from sympatric allosauroid taxa (Samathi, Chanthasit & Sander, 2019). This potential difference in niches is also supported by our results, which illustrate a smaller median size class for megaraptorans compared to allosauroids, followed by an increase in median size class like tyrannosauroids after their extinction and apex predatory taxa like *Maip macrothorax* and *Joaquinraptor casali* (Table 2) (Fig. 4C) (Porfiri et al., 2018; Novas et al., 2019; Lamanna et al., 2020, 2022; Aranciaga Rolando et al., 2022; Ibiricu et al., 2025).

Cau (2024) utilized size classes from Holtz (2021) and found a lower total number of size classes present in tyrannosaur-dominated ecosystems than in non-tyrannosaur megatheropod-dominated ecosystems. Cau (2024) also found a difference between relative size classes, with a higher contribution from smaller size classes in tyrannosaurid-dominated environments and a higher contribution from middle size classes in non-tyrannosaur-dominated ecosystems. Our data illustrates a higher median number of missing size classes in tyrannosauroid environments, and both of these hypotheses may be explained in part by ontogenetic niche shifting/ontogenetic dietary shifts (Fig. 3) (Tables 7, 8) (Therrien et al., 2021; Schroeder, Lyons & Smith, 2021; Holtz, 2021; Therrien et al., 2023; Cau, 2024). There is potential evidence for dietary shifts and potential niche shifting in tyrannosaurids, such as *Albertosaurus sarcophagus* and *Gorgosaurus libratus*, which could provide evidence for this shift in *Tyrannosaurus rex* (Therrien et al., 2021; Schroeder, Lyons & Smith, 2021; Therrien et al., 2023). However, future reanalysis may be needed with the resurrection of *Nanotyrannus lancensis* and the new species *Nanotyrannus lethaeus* to see if this alters the statistical results (Longrich & Saitta, 2024; Zanno & Napoli, 2025; Griffin et al., 2025). The significant difference between these allosauroid/megalosauroid and tyrannosauroid environments may relate to potential ontogenetic niche shifting or ontogenetic dietary niche shifting in members of Tyrannosauridae, preventing other clades from establishing themselves in smaller size classes (Therrien et al., 2021; Schroeder, Lyons & Smith, 2021; Holtz, 2021; Therrien et al., 2023). Statistics from this dataset point towards potential differences present between tyrannosauroid and allosauroid/megalosauroid ecosystems (Tables 7, 8) (Fig. 3).

Abelisauroid and abelisauroid/megaraptoran environments show the same median number of missing theropod size classes, and abelisauroid environments have a more restricted upper and lower quartile, and this is likely due to the lower number of data points rather than the abelisauroid/megaraptoran data points (Fig. 3) (File S7). Abelisauroids may still have a higher median number of missing size classes compared to allosauroid/megalosauroid environments, despite localities in North Africa, India, and Madagascar exhibiting multiple distinct ceratosaurian species of different sizes, potentially occupying different niches (Bandyopadhyay et al., 2010; Delcourt, 2018; Longrich et al., 2023).

Taphonomy is a factor to consider when analyzing these size class patterns. Gaps or missing records can impact the results of trends seen. While the fossil record is incomplete, the literature provides evidence to support some of the claims made from the data. It should be noted that while megaraptorans are the least widespread currently, this clade may exhibit a greater spatial expansion in the future as new taxa are described, and the same can be stated for the other clades as well. This is a limitation of the study at present. Another instance of potential taphonomic bias includes the smaller median size class for tyrannosauroids during the Cenomanian-Turonian time bin, which could be due to fewer fossils found from this clade. Fewer relative terrestrial formations deposited at this time means this would impact our results, yet evidence pointed to no potential change in diversification rates, meaning it is possible the tyrannosauroids weren’t necessarily more diverse or more rare, and our results are representative of an accurate signal (Mannion, 2024). It is possible that the median size class for tyrannosauroids during this time bin was larger, and while this is a limitation of the study, there is evidence from our results and the literature of a general trend of smaller tyrannosauroids prior to the KTM, followed by an increase in size as they take over the apex predator niche in Asiamerica (Holtz, 2021).

Similar lower specific numbers of known ceratosaurians, tyrannosauroids, and allosauroids compared to megalosauroids in the Middle Jurassic may be due to taphonomy, with megalosauroids preferring coastal environments that led to higher rates of preservation over these other clades that may have lived in other habitats, such as inland environments (Rauhut, Hübner & Lanser, 2016). Large-scale trends seen in our data, such as the shift in median body size associated with megaraptorans and tyrannosauroids occupying the apex predator niche, provide evidence for this hypothesis (Figs. 4C, 4D) (Table 2) (Holtz, 2021; Lamanna et al., 2022). Our results and the significant differences between specific clades also support potential size, morphological, or potential habitat differentiation among taxa (Fig. 4) (Tables 3, 4) (Farlow & Pianka, 2002; Cau, 2024). While our results support different hypotheses from the literature, clade diversity and thus their median size classes over time are impacted by taphonomic bias and an incomplete fossil record, meaning this may impact some of the results of this study.

## Conclusions

Statistical analysis indicates there is a significant difference between the median size classes for four pairs of clades containing obligate faunivorous megatheropods (Allosauroidea-Ceratosauria, Allosauroidea-Megaraptora, Allosauroidea-Tyrannosauroidea, along with Megalosauroidea-Ceratosauria). This implies there is a potential series of ecological connections between these clades through time, both before and after the extinction of Allosauroidea and Megalosauroidea during the Cenomanian-Turonian, when the surviving clades take over the apex predator niche in different landmasses. There is a lack of significant differences in median size class between these three surviving clades. There is a significant difference in the median number of missing size classes between allosauroid/megalosauroid-dominated ecosystems and tyrannosauroid-dominated ecosystems from previous data analyzed. Future work can compare these trends in median size class with morphology, diet, and possible habitat differentiation to unravel potential interconnected mechanistic relationships between these variables in megatheropod evolution.

## Supplemental Information

10.7717/peerj.21007/supp-1 Supplemental Information 1 Original occurrence matrix used for the analysis.

10.7717/peerj.21007/supp-2 Supplemental Information 2 Theropod Analysis Matrix. Mass and length estimates, size class, age and stage of the genera and species along with time bins.

10.7717/peerj.21007/supp-3 Supplemental Information 3 RStudio Analysis Code. RStudio code used for producing the plots in the analysis.

10.7717/peerj.21007/supp-4 Supplemental Information 4 Clade Temporal Diversity. Number of species per time bin for each clade.

10.7717/peerj.21007/supp-5 Supplemental Information 5 Clade Spatial Diversity. Number of species per clade for each landmass.

10.7717/peerj.21007/supp-6 Supplemental Information 6 Theropod size class medians. The median size class for each clade in each time bin. Single taxon occurrences for a clade during a time bin were marked as NA.

10.7717/peerj.21007/supp-7 Supplemental Information 7 Modified Theropod Guild Data. Modified from a dataset in Holtz (2021) to reproduce a box and whisker plot detailing the median number of missing size classes in environments dominated by different types of apex theropod predators.

10.7717/peerj.21007/supp-8 Supplemental Information 8 Size Class Stats. Statistical analysis on median size class between clades.

10.7717/peerj.21007/supp-9 Supplemental Information 9 Additional Statistical Results of File S7 Data. Statistical results one of our authors (TRH) found for the dataset from File S7 using the R program PAST 4.15 (2023) (Hammer, Harper & Ryan, 2001).

10.7717/peerj.21007/supp-10 Supplemental Information 10 Clade species count per time bin. Each clade (Ceratosauria, Allosauroidea, Megalosauroidea, Megaraptora, and Tyrannosauroidea) and its measured number of species are partitioned by the six discrete time bins. Abbreviations: Hett.-Pliens. =Hettangian-Pliensbachian Ages, Toar.-Call.= Toarcian-Callovian Ages, Oxfor.-Tith.= Oxfordian-Tithonian Ages, Berr.-Alb. = Berriasian-Albian Ages, Ceno.-Tur.= Cenomanian-Turonian Ages, Con.-Maas. =Coniacian- Maastrichtian Ages.

10.7717/peerj.21007/supp-11 Supplemental Information 11 Clade species count per landmass. Each clade (Ceratosauria, Allosauroidea, Megalosauroidea, Megaraptora, and Tyrannosauroidea) and its measured number of species are partitioned by their landmasses.

10.7717/peerj.21007/supp-12 Supplemental Information 12 Total global clade distribution. Each clade (Ceratosauria, Megalosauroidea, Allosauroidea, Megaraptora, and Tyrannosauroidea) is represented by convex hulls, with points based on latitude/longitude and calculated paleolatitude/paleolongitude coordinates of the type specimen for each species. The maps in the left column(A, C, E, G, I, K) use paleocoordinates and paleogeography, and the maps in the right column (B, D, F, H, J, L) use modern coordinates and the modern world geography. The plots use two axes and focus on showing the spatial extent of each clade across their time bins. *(A-B)* Hettangian-Pliensbachian. *(C-D)* Toarcian-Callovian. *(E-F)* Oxfordian-Tithonian. *(G-H)* Berriasian-Albian. *(I-J)* Cenomanian-Turonian. *(K-L)* Coniacian-Maastrichtian. The paleomaps for each time bin are estimated for 198, 172,151,122, 95, and 78 million years ago, respectively.

10.7717/peerj.21007/supp-13 Supplemental Information 13 Obligate faunivorous megatheropods during the Cretaceous. The center of the piece features the widespread carcarodontosaurids and spinosaurs associated with global Cenomanian fauna such as found in the Kem Kem group. As the two clades were important to theropod ecology, the right and left sides show the different routes faunas in Laurasia and Gondwana took after the Cretaceous Thermal Maximum and the extinction of allosauroids and megalosauroids. The left shows Laurasian ecosystems dominated by tyrannosaurs and ornithischians, whereas the right shows Gondwanan ecosystems dominated by abelisaurids, megaraptorids and titanosaurs. Created by Sergey Krasovskiy and Pedro Salas.

## Additional Information and Declarations

### Data Availability

The following information was supplied regarding data availability:

The raw data and code are available in the Supplemental Files.
