# Supplementary figures and images for: Obligate faunivorous megatheropod size class patterns across the Jurassic-Cretaceous Periods
Source: PeerJ. 2026 Apr 9;14:e21007. doi: 10.7717/peerj.21007 (PMC13070321; doi:10.7717/peerj.21007)

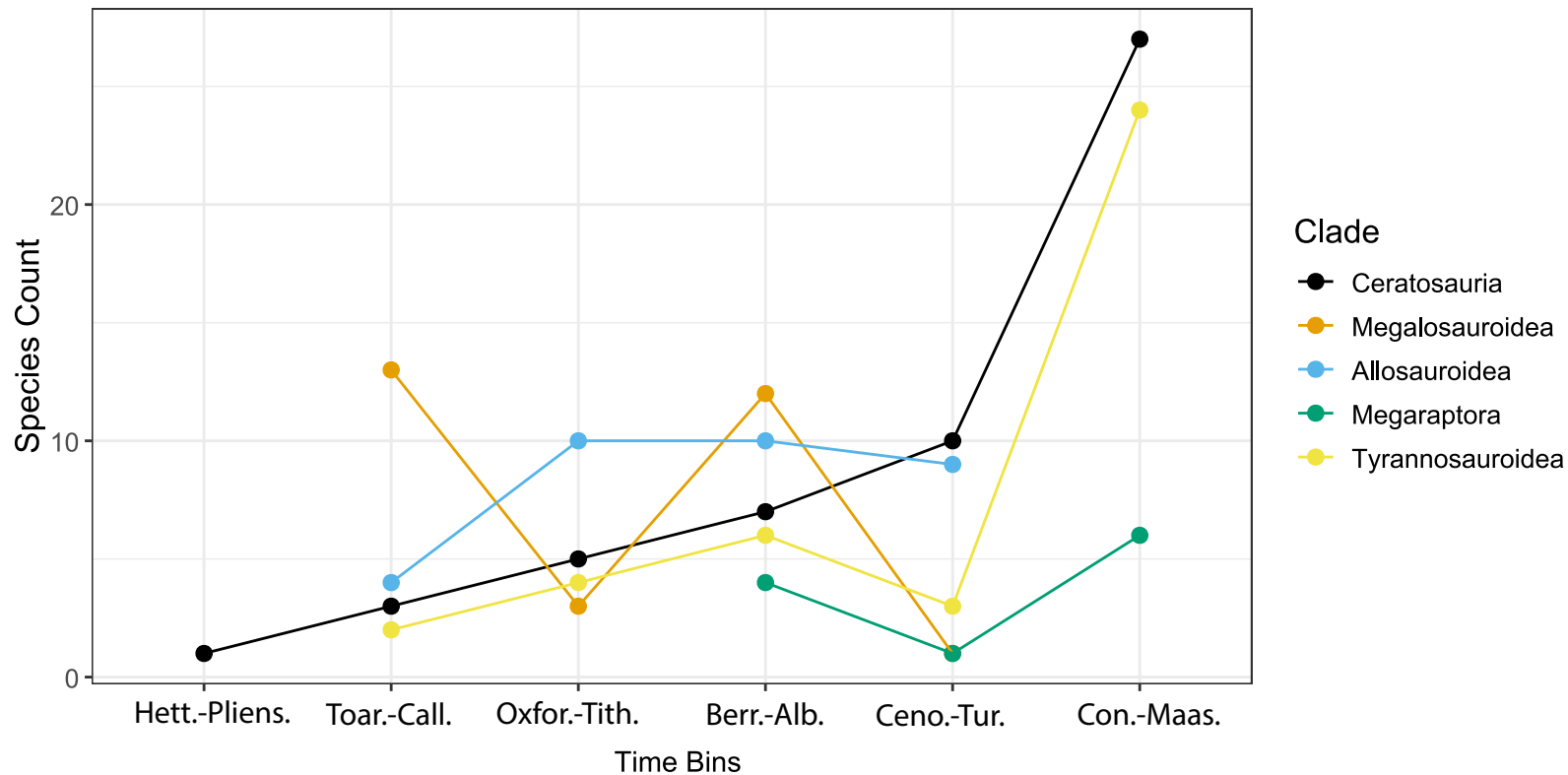

Supplement: Supplemental Information 10 — Each clade (Ceratosauria, Allosauroidea, Megalosauroidea, Megaraptora, and Tyrannosauroidea) and its measured number of species are partitioned by the six discrete time bins. Abbreviations: Hett.-Pliens. =Hettangian-Pliensbachian Ages, Toar.-Call.= Toarcian-Callovian Ages, Oxfor.-Tith.= Oxfordian-Tithonian Ages, Berr.-Alb. = Berriasian-Albian Ages, Ceno.-Tur.= Cenomanian-Turonian Ages, Con.-Maas. =Coniacian- Maastrichtian Ages. [file peerj-14-21007-s010.pdf]

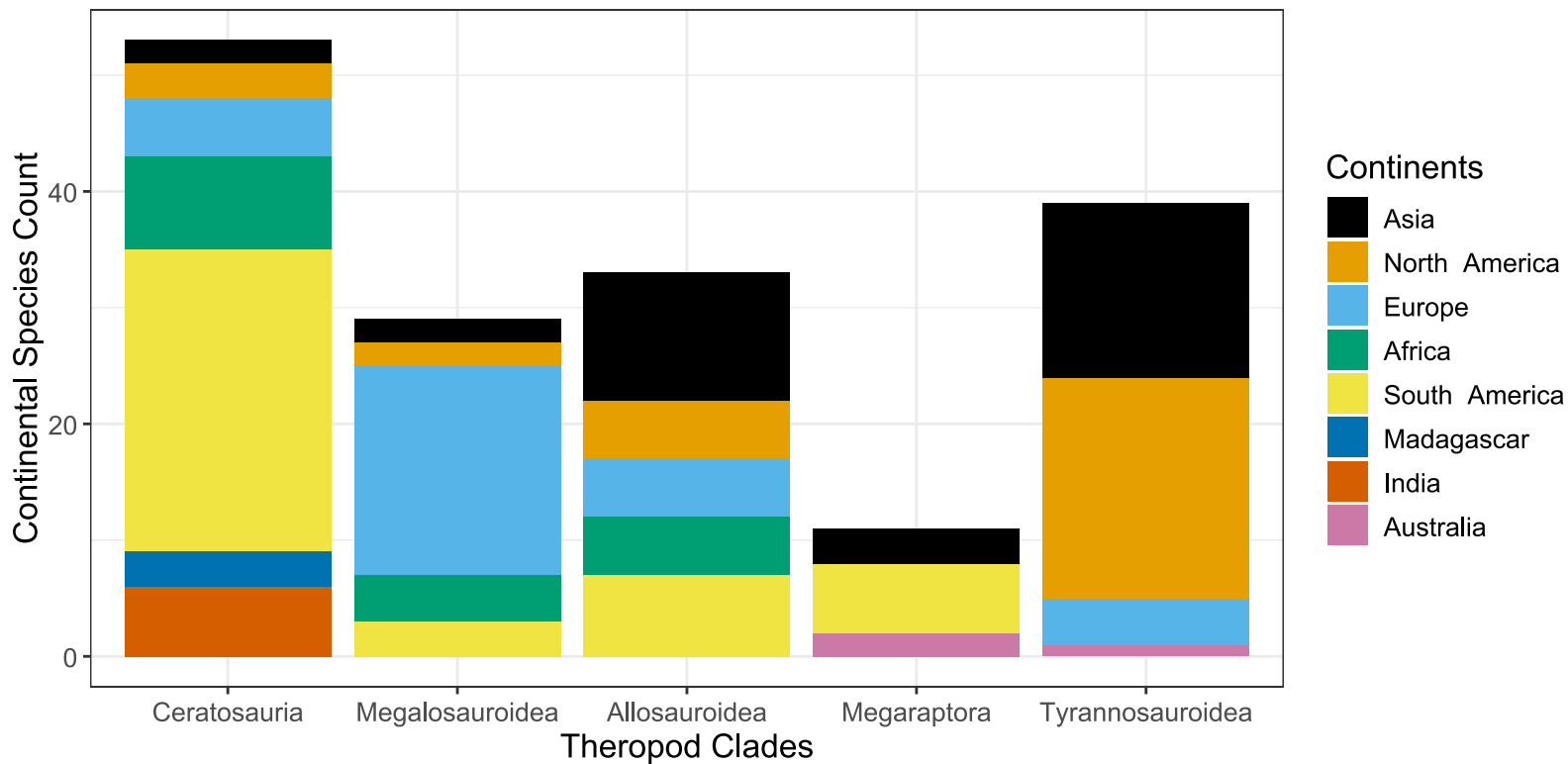

Supplement: Supplemental Information 11 — Each clade (Ceratosauria, Allosauroidea, Megalosauroidea, Megaraptora, and Tyrannosauroidea) and its measured number of species are partitioned by their landmasses. [file peerj-14-21007-s011.pdf]

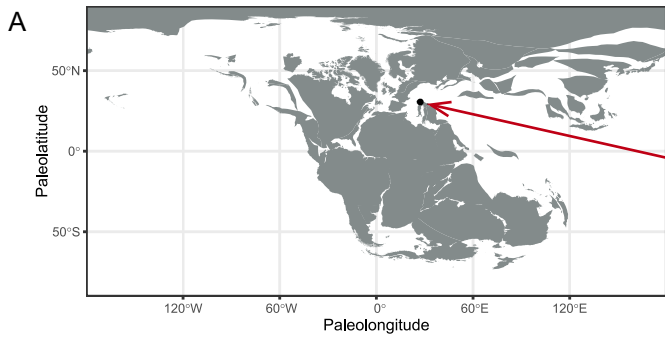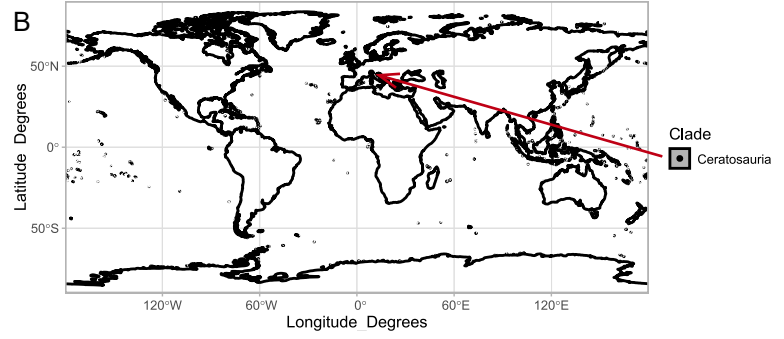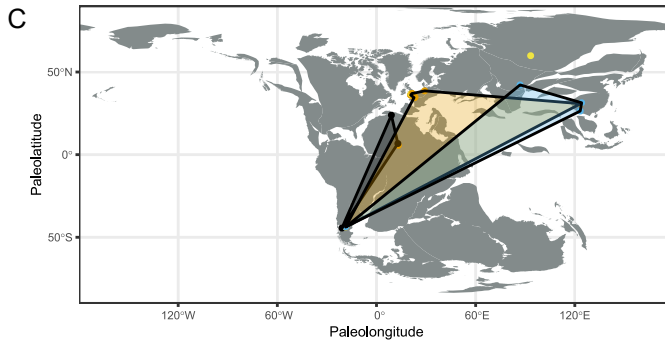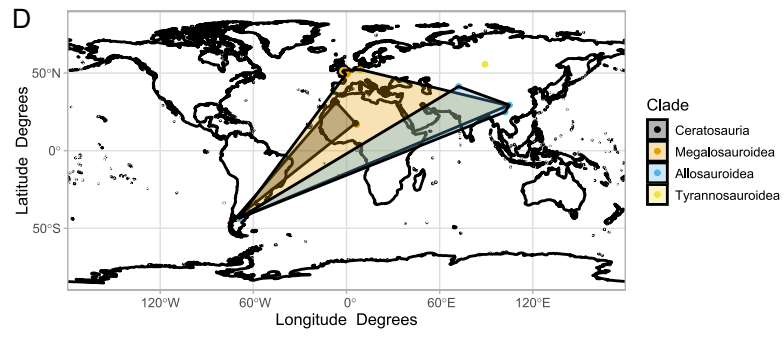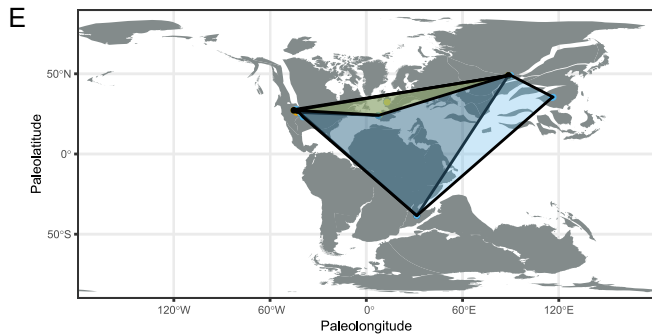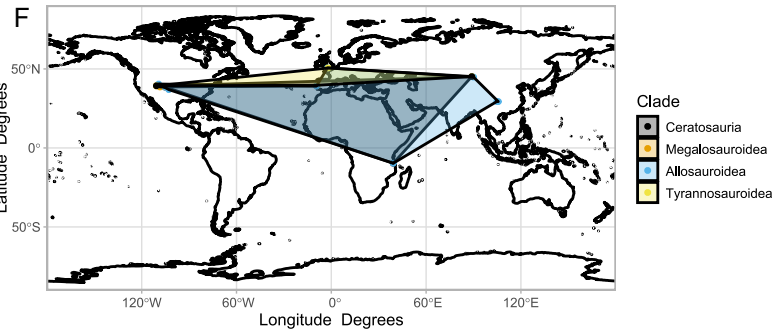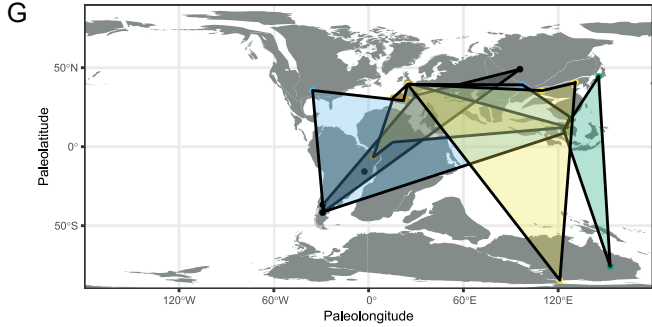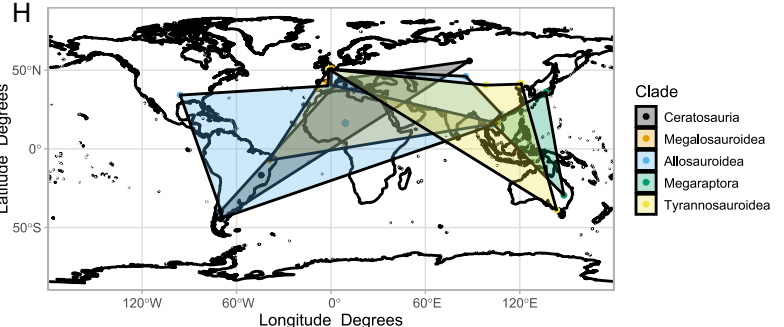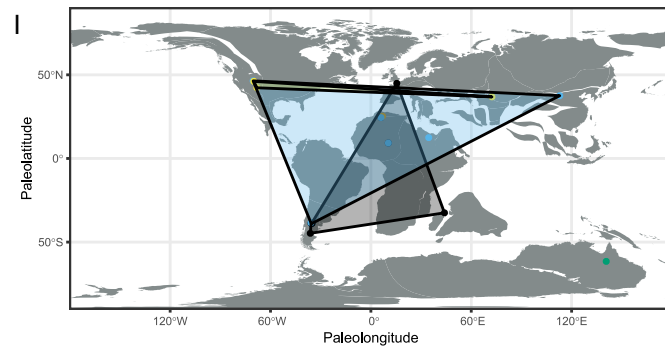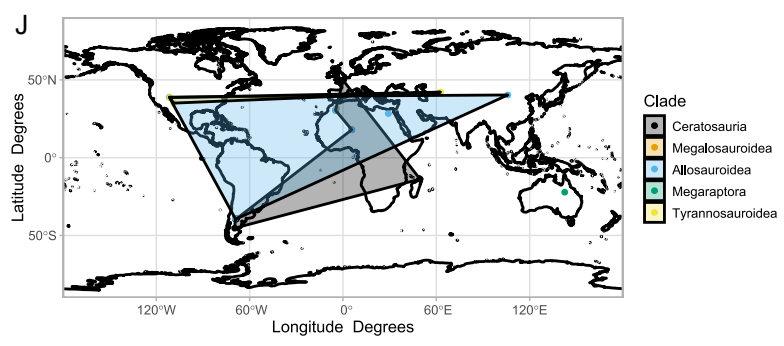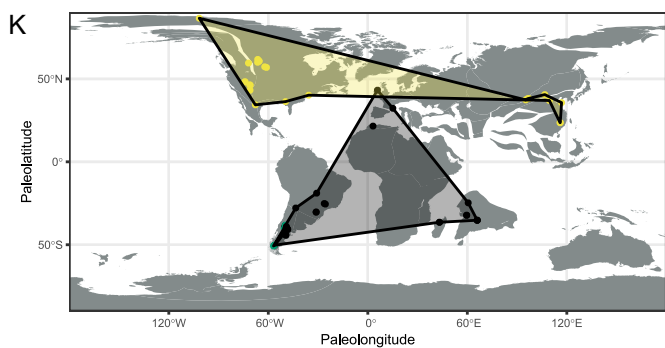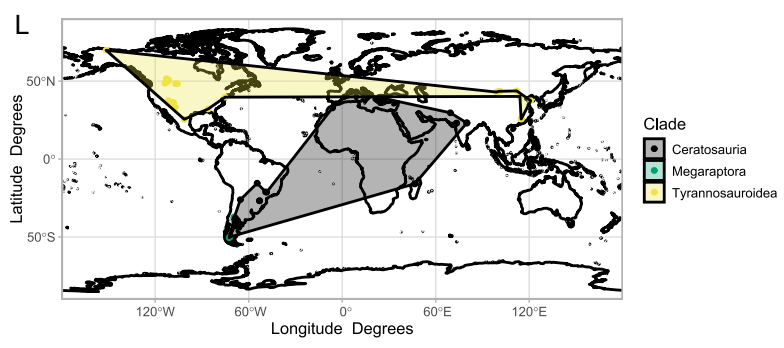

Supplement: Supplemental Information 12 — Each clade (Ceratosauria, Megalosauroidea, Allosauroidea, Megaraptora, and Tyrannosauroidea) is represented by convex hulls, with points based on latitude/longitude and calculated paleolatitude/paleolongitude coordinates of the type specimen for each species. The maps in the left column(A, C, E, G, I, K) use paleocoordinates and paleogeography, and the maps in the right column (B, D, F, H, J, L) use modern coordinates and the modern world geography. The plots use two axes and focus on showing the spatial extent of each clade across their time bins. (A-B) Hettangian-Pliensbachian. (C-D) Toarcian-Callovian. (E-F) Oxfordian-Tithonian. (G-H) Berriasian-Albian. (I-J) Cenomanian-Turonian. (K-L) Coniacian-Maastrichtian. The paleomaps for each time bin are estimated for 198, 172,151,122, 95, and 78 million years ago, respectively. [file peerj-14-21007-s012.pdf]

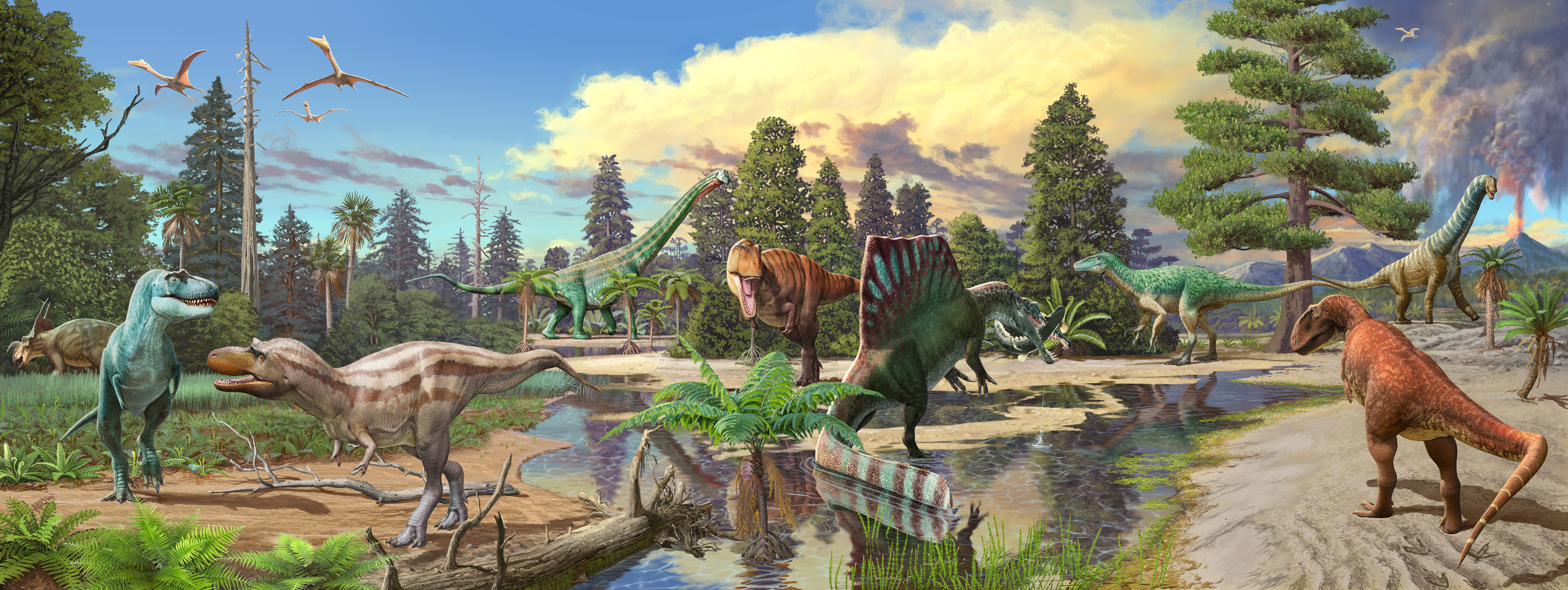

Supplement: Supplemental Information 13 — The center of the piece features the widespread carcarodontosaurids and spinosaurs associated with global Cenomanian fauna such as found in the Kem Kem group. As the two clades were important to theropod ecology, the right and left sides show the different routes faunas in Laurasia and Gondwana took after the Cretaceous Thermal Maximum and the extinction of allosauroids and megalosauroids. The left shows Laurasian ecosystems dominated by tyrannosaurs and ornithischians, whereas the right shows Gondwanan ecosystems dominated by abelisaurids, megaraptorids and titanosaurs. Created by Sergey Krasovskiy and Pedro Salas. [file peerj-14-21007-s013.jpg]
